# Inferior Vena Cava Thrombosis in a Patient with Factor V Leiden Syndrome Presenting with Scrotal Pain

**DOI:** 10.1155/2023/6234371

**Published:** 2023-09-25

**Authors:** Helmy Elhag, Fadl Al-Tairy, Mohammed Shakeeb Dahdaha, Ahmed Shaeshaa, Yavuz Yigit

**Affiliations:** ^1^Department of Emergency Medicine, Hamad Medical Corporation, Doha, Qatar; ^2^Department of Emergency Medicine, Lincoln County Hospital, Lincolnshire, UK; ^3^Blizard Institute, Queen Mary University, London, UK

## Abstract

Thrombosis in the inferior vena cava (IVC) is a rare but serious condition that can lead to significant morbidity and mortality. We present a case report of a 39-year-old male who presented to the emergency department with right flank pain that had progressed to severe back pain, bilateral flank pain, scrotal pain, and leg pain over the course of two days. The pain was severe enough to affect his daily activities. Laboratory investigations revealed a D-dimer level of 17 ng/mL, creatinine level of 110 *µ*mol/L, and a white blood cell count of 10 × 10^9^/L with a CRP level of 5 mg/L. Urine analysis was positive for blood. Doppler ultrasound of both legs showed deep vein thrombosis extending from the external iliac veins to the distal veins of both legs. Further investigation with computed tomography of the abdomen revealed a large thrombus in the distal vena cava extending to the renal artery and both external and internal iliac veins. The patient was diagnosed with Factor V Leiden syndrome based on genetic testing, which revealed a heterozygous mutation in the F5 gene. He was successfully treated with low molecular weight heparin and warfarin, and after five days of hospitalization, he was discharged with warfarin for long-term anticoagulation. This case report emphasizes the importance of considering IVC thrombosis in patients with a constellation of symptoms, including scrotal pain, and the role of genetic testing in identifying underlying hypercoagulable states.

## 1. Introduction

Thrombosis in the inferior vena cava (IVC) is a rare but serious condition that can lead to significant morbidity and mortality. It is typically associated with vague symptoms such as abdominal pain, back pain, flank pain, leg pain, and swelling, which can vary in severity depending on the level of the thrombus in the vein. The causes of IVC thrombosis can vary, ranging from hypercoagulable states and tumors to pregnancy and congenital or acquired anomalies in the anatomy of the vein [1, 2].

Although relatively uncommon, IVC thrombosis is associated with a higher risk of mortality than the deep vein thrombosis (DVT) alone, making early diagnosis and treatment essential for optimal patient outcomes [3]. In this case report, we describe a patient with thrombosis in the IVC who presented with a constellation of symptoms and underwent successful treatment with anticoagulation therapy.

## 2. Case Report

A 39-year-old male presented to the emergency department with right flank pain that had progressed to severe back pain, bilateral flank pain, scrotal pain, and leg pain over the course of two days. The pain was severe enough to affect his daily activities. He had no significant medical history and had been treated with analgesics at a local health center prior to presenting to the emergency department. Given analgesics, the patient's back pain decreased. However, he reported that his bilateral testicular pain and bilateral thigh pain became severe. His vital signs were BP 120/80 mmHg, pulse 130 beats/min, RR 20 breaths/min, and temperature 37°C. The leg raising test was negative, and the neurovascular bundle was intact. Scrotal exam was unremarkable. Since both the cremasteric reflex and Phren's sign yielded negative results, an official scrotal Doppler ultrasound was not performed. There was no lower limb edema or swelling. Laboratory investigations revealed a D-dimer level of 17 mg/L (normal range: 0–0.49 mg/L), creatinine level of 124 *µ*mol/L (normal range: 64–110 *µ*mol/L), and a white blood cell count of 10 × 10^3^/L (normal range: 4–10 10^3^/L) with a CRP level of 5 mg/L (normal range: 0–5 mg/L). The patient's prothrombin time showed a slight elevation at 13.8 seconds (normal range: 9.4–12.5 seconds), while APTT and INR levels remained within the normal ranges (APTT: 28.4 seconds, normal range: 25.1–36.5 seconds; INR: 1.1, normal range: ≤1.1).

**Figure 1:**
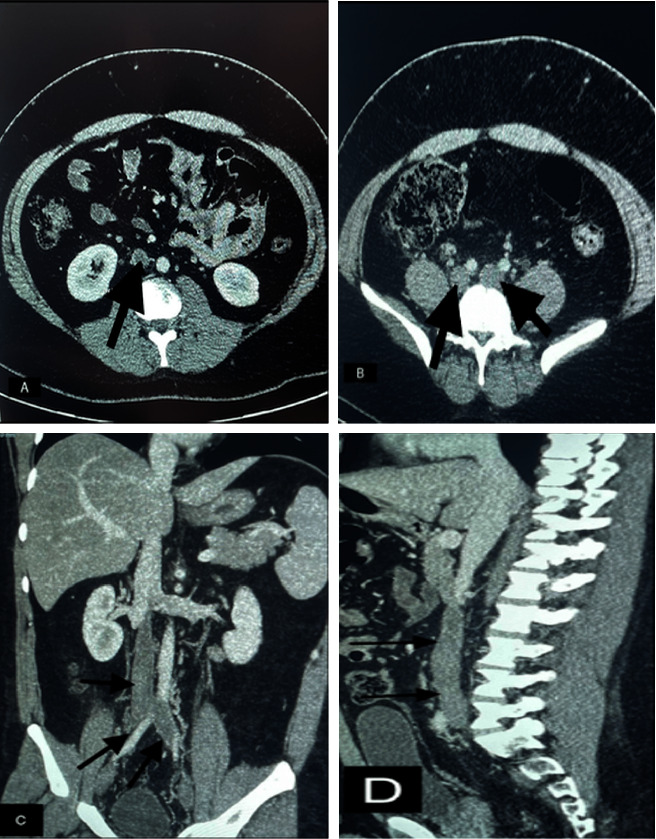
CT abdomen and pelvis with contrast. Large thrombus in the distal vena cava extending to the renal artery and both external and internal iliac veins.

Urine analysis was positive for blood. Bedside abdominal ultrasound showed no significant abnormalities. Doppler ultrasound of both legs showed deep vein thrombosis extending from the external iliac veins to the distal veins of both legs. Further investigation with computed tomography of the abdomen revealed a large thrombus in the distal vena cava extending to the renal artery and both external and internal iliac veins (Figure 1). There was no evidence of renal calculi or other abdominal pathology. Following admission, a comprehensive coagulopathy blood panel was conducted. Protein C levels were in the normal range (89%), but protein S levels were suboptimal (49.9%), likely influenced by the patient's ongoing warfarin sodium therapy. Lupus anticoagulant was not detected, and the patient demonstrated effective anticoagulation with warfarin (ATAC 91%). Our patient, despite the absence of any family history of thrombosis, received a diagnosis of Factor V Leiden syndrome through genetic testing, which unveiled a heterozygous mutation in the F5 gene. He was started on low molecular weight heparin and warfarin, and after five days of hospitalization, he was discharged with warfarin for long-term anticoagulation.

## 3. Discussion

Thrombosis of the inferior vena cava (IVC) is a serious condition that can cause a range of symptoms, making it a challenging diagnosis [4]. This case report described a patient who presented with a variety of symptoms, including severe back pain, flank pain, scrotal pain, and leg pain, which were ultimately attributed to IVC thrombosis. The patient was successfully treated with anticoagulation therapy.

Our case report is unique in which it describes a patient with a large thrombus in the distal vena cava extending to the renal artery and both external and internal iliac veins, which are relatively uncommon locations for IVC thrombosis [5]. In addition, the patient presented with a constellation of symptoms that included scrotal pain, which is an uncommon presentation of IVC thrombosis. The case also emphasizes the importance of genetic testing to identify underlying hypercoagulable states, such as Factor V Leiden syndrome, in patients with IVC thrombosis.

The symptoms of IVC thrombosis can be vague and nonspecific, making the diagnosis challenging [5]. The patient in this case presented with right flank pain that had progressed to severe back pain, bilateral flank pain, scrotal pain, and leg pain over the course of two days. The pain was severe enough to affect his daily activities. These symptoms can be indicative of a variety of conditions, including musculoskeletal pain, urological disorders, and vascular disease, making it difficult to make an accurate diagnosis based on clinical presentation alone [4].

Laboratory investigations, including D-dimer levels, creatinine levels, and a white blood cell count, can help support the diagnosis of IVC thrombosis [6]. In this case, the patient had an elevated D-dimer level and a slightly elevated creatinine level. These laboratory findings are consistent with IVC thrombosis, as D-dimer is a marker of thrombosis, and an elevated creatinine level can be indicative of renal insufficiency, which can occur as a result of IVC thrombosis.

Sudden onset of testicular pain requires immediate evaluation, including a comprehensive medical history, thorough physical examination, urinalysis, and imaging [7, 8]. An urgent situation such as testicular torsion, characterized by a high-riding testicle, demands swift action and confirmation to prevent potential testicular loss [7–9]. Based on our examination and urinalysis, we were able to confidently rule out any potential medical emergencies for our patient. Although less frequent, nonscrotal factors can also trigger testicular pain due to shared nerve pathways (T10–T12, L1-L2, and S2–S4). Conditions such as kidney stones, lower back issues, aortic aneurysms, spinal disc protrusions, hernias, and other abdominal problems that utilize these common nerve routes may manifest as sudden testicular pain [10, 11]. In our case, the patient's testicular pain was likely due to the presence of the thrombus in the iliac veins, which can cause venous congestion and swelling in the scrotum. It is important for clinicians to consider IVC thrombosis in the differential diagnosis when evaluating patients with testicular pain, particularly in patients with known risk factors such as hypercoagulable states.

Bedside abdominal ultrasound is a useful tool for evaluating a wide range of conditions in the acute care setting [12, 13], but it has limitations and may not always provide a definitive diagnosis. If a bedside abdominal ultrasound was performed to evaluate for IVC thrombosis and no abnormal findings were detected, there could be several reasons why this occurred.

Firstly, it is possible that the thrombus was located in a segment of the IVC that was not adequately visualized by the ultrasound exam. The sensitivity of ultrasound for detecting IVC thrombosis can be affected by several factors, including the size and location of the thrombus and the quality of the ultrasound equipment and technique used [5].

In addition, if the thrombus is partially or completely occlusive, there may still be some blood flow within the IVC, which can make it more difficult to detect with ultrasound. Imaging studies, including Doppler ultrasound and computed tomography (CT) scans, can help to confirm the diagnosis of IVC thrombosis [5]. Doppler ultrasound is a noninvasive and cost-effective way to diagnose deep vein thrombosis (DVT), which can be a precursor to IVC thrombosis. In this case, Doppler ultrasound revealed DVT extending from the external iliac veins to the distal veins of both legs. CT scans can provide more detailed information about the location and extent of the thrombus [5]. In this case, a CT scan of the abdomen revealed a large thrombus in the distal vena cava extending to the renal artery and both external and internal iliac veins.

The causes of IVC thrombosis can vary, ranging from hypercoagulable states and tumors to pregnancy and congenital or acquired anomalies in the anatomy of the vein [6]. In this case, the patient was diagnosed with Factor V Leiden syndrome based on genetic testing, which revealed a heterozygous mutation in the F5 gene. Factor V Leiden is a common genetic risk factor for thrombosis, and individuals with this mutation have an increased risk of developing venous thromboembolism (VTE), including IVC thrombosis [14].

Treatment for IVC thrombosis typically involves anticoagulation therapy, which can prevent the thrombus from growing and reduce the risk of pulmonary embolism [6]. The patient in this case was started on low molecular weight heparin and warfarin, and after five days of hospitalization, he was discharged with warfarin for long-term anticoagulation. It is important to note that anticoagulation therapy can increase the risk of bleeding, and careful monitoring is required to ensure that the patient's INR is within the therapeutic range.

## 4. Conclusion

The presented case highlights the challenge of diagnosing IVC thrombosis due to its nonspecific symptoms and emphasizes the importance of considering this condition in the differential diagnosis for patients presenting with back pain, flank pain, scrotal pain, and leg pain. Laboratory investigations, imaging studies, and genetic testing can aid in the diagnosis of IVC thrombosis and identifying underlying hypercoagulable states. Anticoagulation therapy is the mainstay of treatment, but careful monitoring is required to avoid complications, such as bleeding. Clinicians should consider IVC thrombosis in the differential diagnosis for patients presenting with testicular pain, particularly in those with known risk factors. Early recognition and management of IVC thrombosis can prevent life-threatening complications, including pulmonary embolism.

## Data Availability

The data used to support the findings of this study are available from the corresponding author upon request.
